# Assessing Cardiovascular Dysfunction Using Cardiac Strain After Diphenhydramine Overdose

**DOI:** 10.7759/cureus.101242

**Published:** 2026-01-10

**Authors:** George G Kidess, Nathan Roberts, Lauren Harvey, Nigel Bowe, Erin Cuddeback, Abigail R Brackney, Brandtly Yakey, Caroline Dowers, Christopher R Clark

**Affiliations:** 1 Medicine, The University of Chicago Medicine, Chicago, USA; 2 Emergency Medicine, Henry Ford Health System, Detroit, USA

**Keywords:** diphenhydramine overdose, drug overdose, global longitudinal strain, left ventricular systolic dysfunction, lv ejection fraction (lvef)

## Abstract

Diphenhydramine overdose is a dangerous condition that typically leads to symptoms of antimuscarinic toxicity and dysrhythmias. Left ventricular (LV) systolic dysfunction is a manifestation of diphenhydramine toxicity that commonly presents with electrocardiogram (ECG) findings of QRS prolongation. This article presents the case of a patient who was brought to the emergency department (ED) after a diphenhydramine overdose and was showing signs of antimuscarinic toxicity, with an ECG showing no signs of QRS interval prolongation. Point-of-care echocardiography and speckle tracking were performed, which showed a preserved ejection fraction (EF) and reduced global longitudinal strain (GLS), suggestive of subtle LV systolic dysfunction. The patient was treated with intravenous fluids and rivastigmine; his symptoms improved appropriately, and he was discharged the following day. This case report highlights the sensitivity of GLS in unveiling LV systolic dysfunction in patients with preserved EF and a normal ECG, showing its potential utility in patients presenting to the ED with substance overdoses.

## Introduction

Diphenhydramine is a common over-the-counter antihistamine that is used in most age groups to treat seasonal allergies and sleeping difficulties [1]. While diphenhydramine is a relatively safe drug when taken appropriately, an overdose can often be incredibly harmful. The antimuscarinic toxidrome, caused by blockade of muscarinic acetylcholine receptors, leads to central symptoms of delirium and agitation as well as peripheral effects of tachycardia, anhidrosis, and urinary retention [1]. A feared complication of diphenhydramine overdoses is cardiac conduction abnormalities, such as QRS and QTc prolongation, as well as ventricular dysrhythmias, due to inhibition of fast sodium channels and delayed rectifier potassium channels [1-4].

Importantly, rates of diphenhydramine overdose have been rising nationally over the last few decades, with most being caused by suicide attempts, self-harm, or intentional misuse [5]. Severe outcomes from diphenhydramine overdose, including seizures, ventricular dysrhythmias, and the need for intubation, have been increasingly reported, with risk factors for a more severe outcome including acidemia, QRS interval prolongation, and an elevated anion gap [4]. It is also essential to emphasize the importance of incorporating socioeconomic determinants into the discussion of cardiovascular health and overdose, as aspects such as housing instability, lower income, educational status, and insurance coverage, among others, can significantly impact health [6]. This article reports the case of a patient presenting with a diphenhydramine overdose and showing signs of left ventricular (LV) dysfunction as measured by global longitudinal strain (GLS) despite a normal electrocardiogram (ECG).

## Case presentation

**Figure 1:**
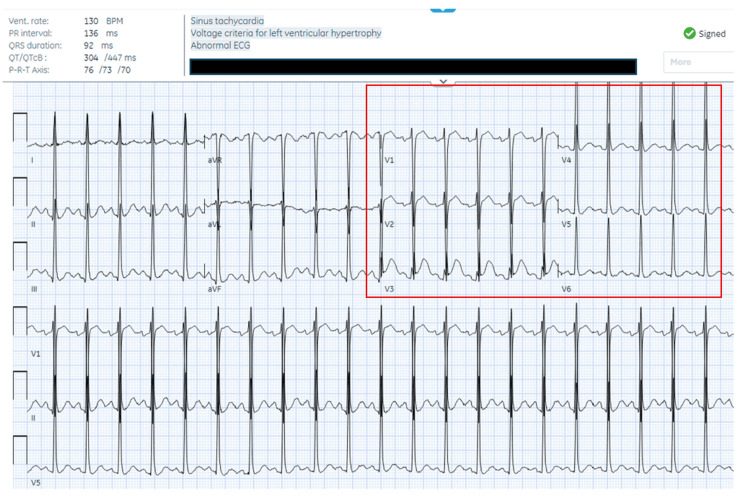
Electrocardiogram on day one in the emergency department, showing sinus tachycardia with signs of left ventricular hypertrophy (highlighted with red box), but no ST elevations or depressions, T-wave inversions, or QRS or QTc prolongation

The patient is a 51-year-old male with a history of bipolar disorder, psychosis, and alcohol use disorder who presented to our urban emergency department (ED) from a substance use recovery residence facility after being found to have an altered mental status. The patient reported ingesting about 10 diphenhydramine 50 mg pills, and about 60 loose pills were found on his person, some of which were crushed. Due to the patient’s altered mental status, collateral history was obtained from the recovery facility, which confirmed that there were low concerns of co-ingestion of other substances. Upon arrival at the ED, his vital signs were: heart rate (HR) 115 bpm, blood pressure 169/113 mmHg, and temperature 37.1°C. Physical examination revealed delirium with a mumbling speech pattern, dry mucous membranes and axilla, and urinary retention. The patient’s urine toxicology screening was positive for tricyclic antidepressants, suspected to be due to cross-reactivity from diphenhydramine ingestion, and negative for alcohol, opioids, amphetamine, and cocaine, among other substances (pertinent lab values can be found in Table 1). An ECG (Figure 1) was also obtained, which did not show any abnormalities; QRS duration was 92 ms, and QTc was 447 ms. Due to concerns of antimuscarinic toxicity, 1 mg of IV lorazepam was administered, and the patient also received 2 L of IV fluids (1 L of normal saline and 1 L of lactated Ringer's), as well as antidotal therapy with one dose of 6 mg rivastigmine. 

**Table 1:** Pertinent lab values WBC, white blood cells; Hb, hemoglobin; CO_2_, carbon dioxide, BUN, blood urea nitrogen

Laboratory investigations	Lab values	Ranges
WBC	19.4	4-11 cell/mL
Hb	14.7	13.5-17.5 g/dL
Platelets	403	140-450 platelets/microL
Sodium	133	135-145 mEq/L
Potassium	3.6 (hemolyzed)	3.6-5.2 mmol/L
Chloride	98	96-106 mEq/L
CO_2_	22	23-29 mEq/L
Anion gap	13	4-12 mEq/L
BUN	16	6-20 mg/dL
Creatinine	1.29	0.7-1.3 mg/dL
Glucose	106	80-120 mg/dL
Opiates	Negative	<300 ng/mL
Cocaine	Negative	<300 ng/mL
Cannabinoids	Negative	<50 ng/mL
Barbiturates	Negative	<200 ng/mL
Benzodiazepines	Negative	<200 ng/mL
Phencyclidine	Negative	<25 ng/mL
Tricyclics	Positive	<300 ng/mL
Amphetamines	Negative	<1000 ng/mL
Acetaminophen	<10	<20 mcg/mL

**Figure 2:**
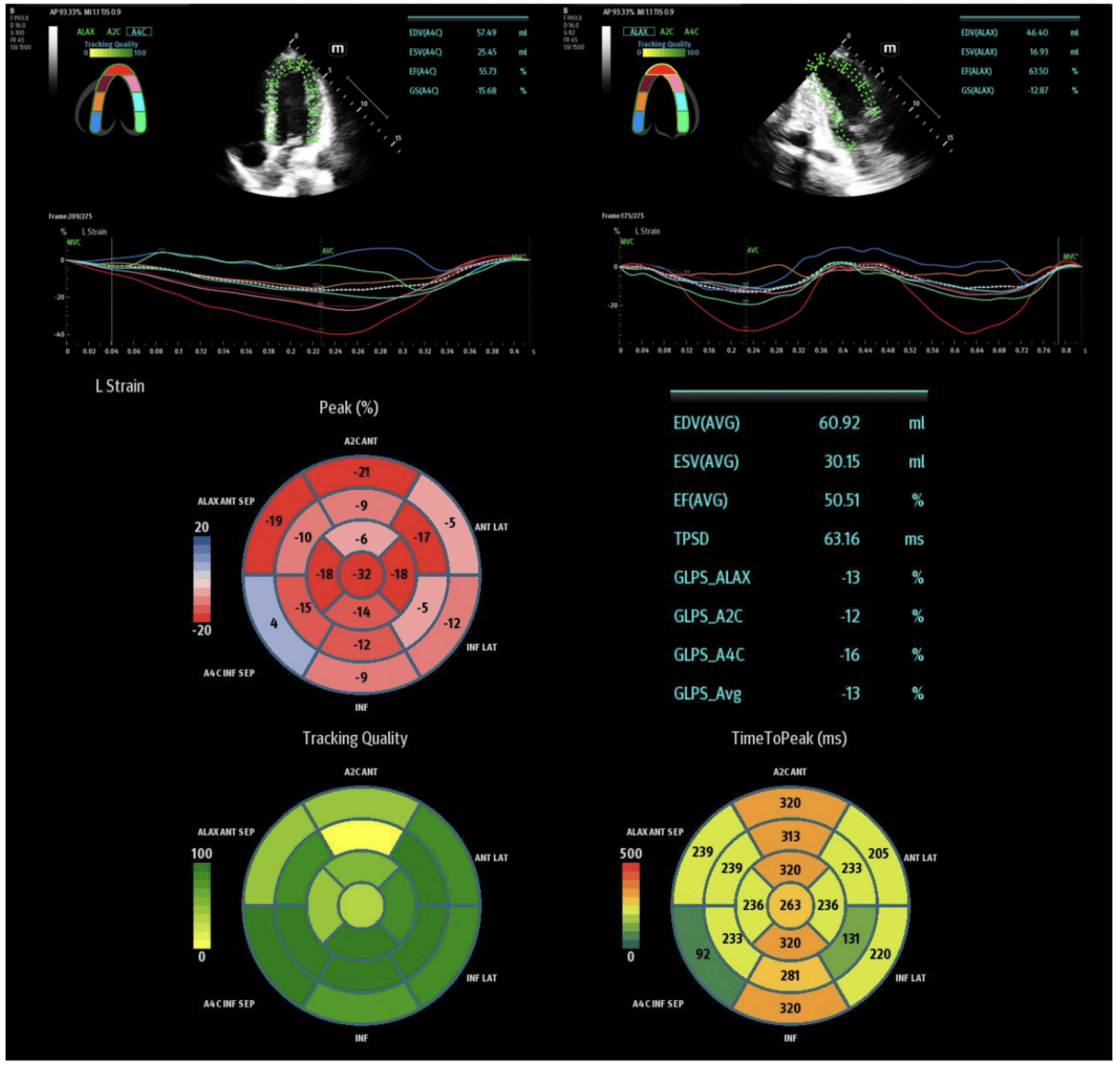
Speckle-tracking echocardiography on day one in the emergency department, showing a reduced global longitudinal strain, preserved ejection fraction, and inferoseptal hypokinesis in the bull's-eye diagram (top left)

**Figure 3:**
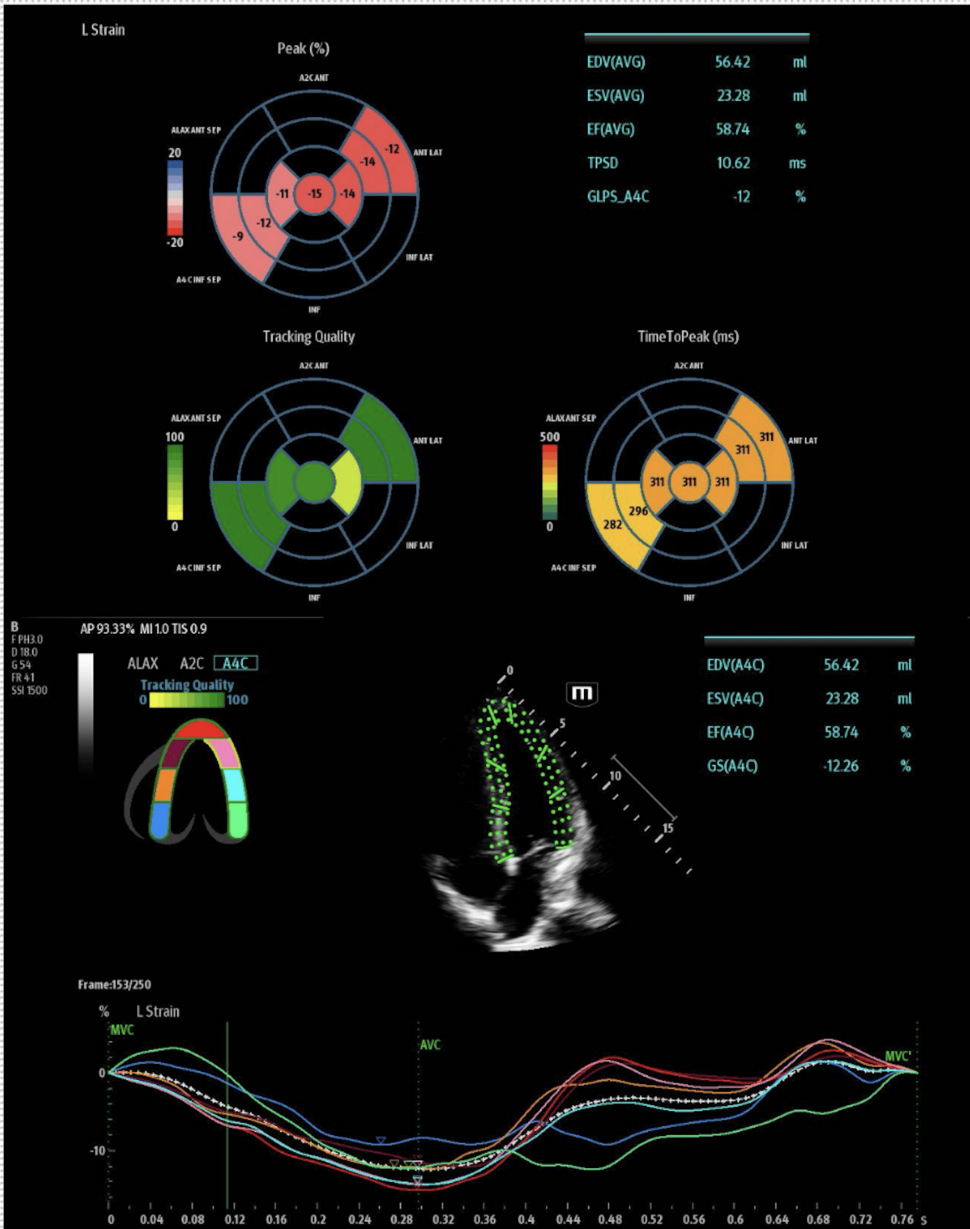
Limited speckle-tracking echocardiography on day two in the emergency department, showing a reduced global longitudinal strain and preserved ejection fraction

After the patient was stabilized with the above therapies, a point-of-care ultrasound (POCUS) echocardiogram was obtained using a Mindray TEX ultrasound system (Mindray, Mahwah, NJ, USA), which showed a preserved ejection fraction (EF) with no wall motion abnormalities as observed by the operator. A speckle-tracking algorithm was used to measure the GLS of the patient’s left ventricle, which showed a reduced average GLS of -13%, despite a relatively normal EF of 50.51%. A bullseye diagram was also obtained, which showed hypokinesis of the inferoseptal wall segment. On the patient’s second day in the ED, a limited POCUS echocardiogram with speckle tracking was obtained, which continued to show reduced GLS (-12.26%) with a preserved EF (58.74%). The bullseye diagram on the second day showed that the inferoseptal hypokinesis that was present on the previous day appeared to have resolved. Representative images of speckle tracking and bullseye diagrams for day one and day two in the ED can be seen in Figure 2 and Figure 3, respectively.

As the patient was being treated, his mentation, tachycardia, and hypertension improved. He was cooperative and reported that he ingested and smoked variable amounts of pills for recreational purposes and had no intention of harming himself. He also confirmed sole ingestion of diphenhydramine and denied other co-ingestions. He was assessed by the psychiatry team, who confirmed that he had no suicidal ideations or intent, and he was then discharged to the substance use recovery residency facility.

## Discussion

Various studies have shown that QRS prolongation is a predictor of severe cardiac complications, such as dysrhythmias and cardiac arrest, for patients with diphenhydramine overdose [2,7,8]. While the cardiotoxic effects of diphenhydramine toxicity are well established secondary to inhibition of fast sodium channels and delayed rectifier potassium channels [1-4], to our knowledge, this case report is the first in the literature reporting sonographic findings of LV dysfunction after diphenhydramine overdose despite a normal ECG. Another case reported by Wang and colleagues described a patient who experienced Takotsubo cardiomyopathy with a reduced EF after overdosing on diphenhydramine, with his EF promptly recovering the next day prior to discharge [9]. Interestingly, in our case, the patient presented with a preserved EF, and LV systolic dysfunction was only unveiled when measuring GLS. GLS is an estimate of LV systolic function that uses speckle-tracking echocardiography to measure the percentage of myocardial fiber shortening throughout the cardiac cycle [10]. Various studies have shown that GLS may have a higher sensitivity for LV dysfunction than EF, as it can often detect signs of LV systolic dysfunction prior to a reduction in EF [11]. GLS has also been shown in previous studies to be effective in differentiating other cardiac diseases, such as ST-elevation myocardial infarction (STEMI) and stress cardiomyopathy [12]. Importantly, GLS has also been correlated with clinical outcomes, with one study showing that among patients admitted with immune-checkpoint inhibitor myocarditis, a lower GLS was strongly correlated with major adverse cardiovascular outcomes (MACE) on follow-up post-discharge regardless of EF [13]. With respect to our case, this suggests that our patient might have a higher risk of MACE despite having a normal EF. 

Given these benefits, GLS has been increasingly utilized as a marker of cardiac damage after exposure to cardiotoxic medications [11]. Various studies have shown its superior capacity to detect LV dysfunction in patients treated with chemotherapy, such as anthracyclines, patients with rheumatological disorders treated with disease-modifying therapies, and patients with chronic kidney disease [11]. GLS has been utilized to a lesser extent with respect to drug overdoses, although some animal studies suggest that it might have some benefits [14]. One interesting finding in our patient was the presence of inferoseptal hypokinesis on his first day in the ED that appeared to have resolved on the second day. While this might suggest a transient, discrete ventricular dysfunction, it is essential to highlight that the various limitations of GLS, including inter-operator variability, adequate tracking quality, and differences in speckle-tracking algorithms by vendor, might also lead to inaccuracies, and that these findings need to be interpreted with caution [11]. This could be a potential limitation in this case, as the two POCUS echocardiograms and GLS were obtained by different operators. Additionally, in cases of transient systolic dysfunction, GLS appears to recover after a specified time, similarly to EF. In one study, patients admitted with anaphylaxis showed a significantly reduced GLS immediately after the reaction, which recovered within six weeks [15]. The GLS in our patient continued to be reduced on the second day of admission, which might suggest that the recovery in systolic function might occur later in the disease course. There was no complete echocardiogram previously or during this admission to which to compare findings.

## Conclusions

Diphenhydramine overdoses are becoming more common and are associated with severe outcomes, including antimuscarinic toxicity and cardiac dysrhythmias. This case report presents a rare presentation of LV systolic dysfunction on echocardiography despite a normal ECG and normal EF in a patient with a diphenhydramine overdose. This case emphasizes the utility of GLS as a sensitive measure of LV systolic dysfunction, highlighting its potential in the ED as an early measure of xenobiotic-induced cardiotoxicity, especially with recent advancements in the use of artificial intelligence in echocardiography to automatically measure LV strain.
